# Ability of Procalcitonin to Discriminate Infection from Non-Infective Inflammation Using Two Pleural Disease Settings

**DOI:** 10.1371/journal.pone.0049894

**Published:** 2012-12-12

**Authors:** Fiona J. McCann, Stephen J. Chapman, Wai Cho Yu, Nick A. Maskell, Robert J. O. Davies, Y. C. Gary Lee

**Affiliations:** 1 Oxford Centre for Respiratory Medicine and University of Oxford, Oxford, United Kingdom; 2 Centre for Asthma, Allergy and Respiratory Research, School of Medicine and Pharmacology, University of Western Australia, Perth, Australia; 3 Department of Medicine, Princess Margaret Hospital, Hong Kong, Special Administrative Region, People's Republic of China; 4 North Bristol Lung Centre, Southmead Hospital, Bristol, United Kingdom; Statens Serum Institute, Denmark

## Abstract

Procalcitonin has been shown to be useful in separating infection from non-infective disorders. However, infection is often paralleled by tissue inflammation. Most studies supporting the use of procalcitonin were confounded by more significant inflammation in the infection group. Few studies have examined the usefulness of procalcitonin when adjusted for inflammation.

Pleural inflammation underlies the development of most exudative effusions including pleural infection and malignancy. Pleurodesis, often used to treat effusions, involves provocation of intense aseptic pleural inflammation. We conducted a two-part proof-of-concept study to test the specificity of procalcitonin in differentiating infection using cohorts of patients with pleural effusions of infective and non-infective etiologies, as well as subjects undergoing pleurodesis.

**Methods:**

We measured the blood procalcitonin level (i) in 248 patients with pleural infection or with non-infective pleural inflammation, matched for severity of systemic inflammation by C-reactive protein (CRP), age and gender; and (ii) in patients before and 24–48 hours after induction of non-infective pleural inflammation (from talc pleurodesis).

**Results:**

1) Procalcitonin was significantly higher in patients with pleural infection compared with controls with non-infective effusions (n = 32 each group) that were case-matched for systemic inflammation as measured by CRP [median (25–75%IQR): 0.58 (0.35–1.50) *vs* 0.34 (0.31–0.42) µg/L respectively, *p* = 0.003]. 2) Talc pleurodesis provoked intense systemic inflammation, and raised serum CRP by 360% over baseline. However procalcitonin remained relatively unaffected (21% rise). 3) Procalcitonin and CRP levels did not correlate. In 214 patients with pleural infection, procalcitonin levels did not predict the survival or need for surgical intervention.

**Conclusion:**

Using a pleural model, this proof-of-principle study confirmed that procalcitonin is a biomarker specific for infection and is not affected by non-infective inflammation. Procalcitonin is superior to CRP in distinguishing infection from non-infective pleural diseases, even when controlled for the level of systemic inflammation.

## Introduction

Procalcitonin, a precursor of calcitonin manufactured in the thyroid, is significantly elevated during many types of bacterial infection [1]–[5]. A rapidly growing body of evidence supports the use of procalcitonin (PCT) to differentiate bacterial from viral or non-infective diagnoses [6], to help risk stratify patients, and to guide antibiotic therapy decisions about initial need for, and optimal duration of, therapy [1]. Despite the promising results, controversies exist over the benefits of procalcitonin over conventional inflammatory markers [2] and a recent meta-analysis concluded that evidence supporting the use of procalcitonin remains inadequate [7]. In certain areas, such as ventilator-associated pneumonia, atypical pneumonia and tuberculosis, the clinical studies on the role of procalcitonin were mixed [1]. Whether procalcitonin predicts patient outcomes in infection remains under investigation.

Infection is usually accompanied by a corresponding degree of inflammation. Most published studies supporting the use of procalcitonin over conventional inflammatory markers (eg C-reactive protein, CRP) have compared procalcitonin in patients with bacterial infection and those with non-infective diseases or other non-bacterial infections [1]. These studies are often confounded by differences in the degree of inflammation between the bacterial infection group and the controls. For example, procalcitonin was significantly higher in bacteremic patients over those with no pathogens cultured [8]; and likewise procalcitonin was higher in severe bacterial infections than in milder viral illnesses [9]. Without adjusting for inflammation, it is not clear if procalcitonin specifically captures signals of infection or of inflammation associated with the more severe infection.

To verify that procalcitonin is indeed a marker of infection, rather than just that of inflammation, procalcitonin needs to be assessed in patients with infective vs non-infective conditions that are matched for their degree of inflammation. The pleural disease setting offers unique opportunities to address this question. This ‘proof-of-principle’ study tested the discriminating value of procalcitonin for infective over non-infective conditions in two pleural disease settings.

First, we compared procalcitonin in case-matched patients with pleural infection and those with non-infective exudative effusions. The patients were matched for their CRP, gender and age. Pleural inflammation underlies the development of exudative pleural effusions. About 40% of exudates are infection-related (parapneumonic or empyema) whereas the others can arise from a variety of pleural or systemic diseases (eg cancer, connective tissue diseases etc) [10]. Patients from either group often have raised inflammatory indices, eg CRP and ESR, as well as pleural fluid leukocytosis, reduced fluid pH and glucose [11]. We hypothesized that procalcitonin can better identify infective pleural inflammation than CRP, and thus the procalcitonin level will be higher in the pleural infection group even over controls matched for their systemic inflammation (by CRP).

Second, to further verify that procalcitonin is not affected by non-infective inflammation, we assayed the blood procalcitonin in patients undergoing talc pleurodesis. Pleurodesis involves iatrogenic induction of an intense non-infective inflammation by injurying the pleura with sclerosants (eg talc) or mechanically (eg abrasion) [12]. Pleurodesis provokes a significant rise in systemic inflammation and associated symptoms, eg fever and chest pain [13]. This provides a unique opportunity to test the ability of procalcitonin in separating non-infective inflammation from infections. We hypothesize that procalcitonin will remain low after pleurodesis whereas other inflammatory markers (eg CRP) will be significantly elevated.

We also compared the ability of procalcitonin and CRP in predicting outcomes of patients with pleural infection.

## Methods

Blood and pleural fluid samples were drawn from the Oxford Pleural Disease Biobank assembled from patients recruited to clinical trials of pleural infection and malignancies [14], [15], which were approved by the local ethics committees, including the Central Oxford Research Ethics Committee and the North West Anglia Health Authority (MREC 98/5/61). All patients gave written consent for the storage of the samples and their use for research studies including assessment of markers of infection/inflammation, such as this presented study. Venous blood samples were collected in blood tubes containing EDTA and centrifuged. The supernatants were stored at −80°C until assay.

### Sample Groups

The study was taken in two parts. The first was to establish if procalcitonin levels were different in patients with pleural infection and those with non-infective (malignant) pleural effusions. Pleural infection was defined as the presence of macroscopic purulent pleural pus, presence of bacteria in the pleural fluid by Gram staining or culture, or a pleural effusion of pH<7.20 in a patient with clinical evidence of infection - as used in the published Multi-center Intrapleural Sepsis Trials [14], [16]. Malignant pleural effusions were defined as effusions in patients with histologically and/or cytologically pleural malignancies confirmed on pleural fluid or biopsies. All case-matching in the study was performed by one investigator (FJM) before, and therefore blinded to, the quantification of procalcitonin.

Blood samples from patients with malignant pleural effusions from a previous unrelated study [17] and from pleural infection patients of the MIST [14] were included. Patients were matched in 1∶1 ratio (n = 32 in each group) for gender, age and serum CRP levels – the latter to allow matching for the degree of systemic inflammation.

In the second part of the study, we assayed the blood procalcitonin level in samples collected from patients who underwent pleurodesis with talc slurry in a previously reported clinical trial [15]. The samples were collected before pleurodesis and at 24–48 hours post-talc instillation.

In addition, we investigate if blood procalcitonin levels predict the outcome in patients with pleural infection. Adverse outcome were defined as need for surgery or death, as conventionally defined in published literature [14]. All patients were included in the Multi-center Intrapleural Sepsis Trial [14]. Procalcitonin levels were compared in patients with adverse outcome, and controls matched for age, gender and CRP (n = 102 in each group) who survived the pleural infection and was successfully treated without surgery.

### Procalcitonin Measurement

Procalcitonin levels were quantified using a commercially-available, validated immunoluminometric assay (Brahms, UK), according to the manufacturer's instructions. Samples were measured in duplicates. Intra-assay variability was assessed by re-analyzing a small number of samples in subsequent batches, to ensure reproducibility.

### Statistical Analysis

Data were presented as mean ± SD if normally distributed or median (25%–75% inter-quantile range) if not normally distributed, unless otherwise stated. Comparisons between groups were performed using the Student's *t*-test and Mann-Whitney Ranked Sum test for parametric and non-parametric data respectively. Comparisons between data pre- and post-pleurodesis and comparisons of groups matched for CRP were performed using paired *t* test or Wilcoxon Signed Rank test in parametric and non-parametric data respectively. Non-parametric data were log transformed before linear regression was assessed using Pearson's correlation test. The above analyses were performed using SigmaStat 3.0 (San Jose, CA, USA). Receiver Operator Characteristics (ROC) analyses were performed by a computer program (SPSS, Chicago, IL, USA). A *p*-value of <0.05 was regarded as statistically significant.

## Results

### Procalcitonin was significantly higher in pleural infection than in non-infective controls matched for systemic inflammation

For the first part of the study, patients with pleural infection and their matched cohort with non-infective (malignant) pleural effusions were compared; their demographics are shown in Table 1.

**Table 1:** Characteristics of the patients with pleural infection matched for CRP to those undergoing pleurodesis for malignant pleural diseases.

	Infective (Pleural Infection) Effusion	Non-infective (Malignant) Effusion
**n = **	32	32
**Age**	66.9±14.7	67.3±14.4
**Sex (M∶F)**	13∶19	13∶19
**CRP**	64.3±59.1	64.0±62.1
**Purulent vs non purulent**	26 ∶ 6	
**Type of underlying malignancy**	Not applicable	Ovary 13%
		Breast 25%
		Lung 34%
		Mesothelioma 9%
		Others 19%

**Figure 1:**
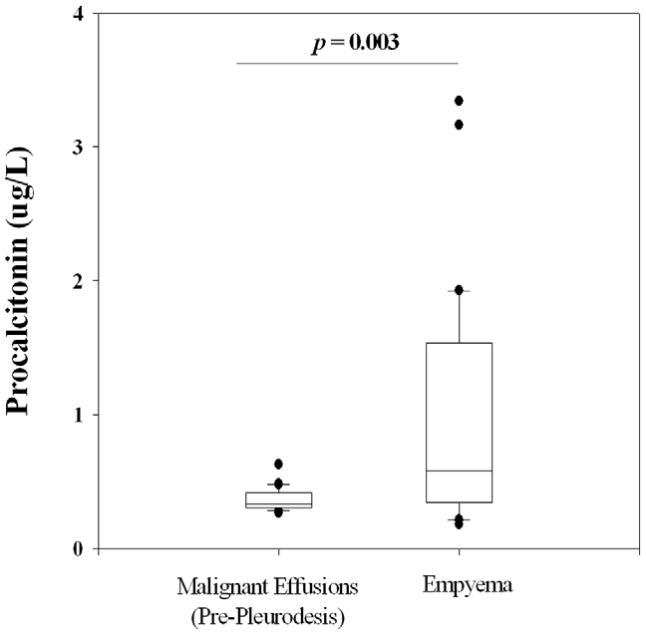
In two groups of patients matched for CRP levels, procalcitonin levels in blood were significantly higher in patients with pleural infection than in those with non-infectious (malignant) pleural effusion. * One outlier in the non-infective group lay outside the margins of the graph and was not shown. (Measurement of procalcitonin in two samples failed and were excluded resulting in a final number of n = 30 in each group; Wilcoxon Signed Rank test).

Procalcitonin levels were significantly higher in the pleural infection group than those with non-infective (malignant) pleural effusions, even when case-matched for their CRP, age and gender. The median procalcitonin levels (25–75 IQR) were 0.58 (0.35–1.50) vs 0.34 (0.31–0.42) µg/L respectively, *p* = 0.003, Fig. 1. ROC analysis revealed that procalcitonin can differentiate the cohort of patients with pleural infection from malignant effusions group with an area under curve (AUC) of 0.804 (95% CI 0.734–0.874) and 0.685 (95% CI 0.589–0.782) for the patients before and after pleurodesis respectively.

### Procalcitonin remained stable even in the presence of intense non-infective inflammation

**Figure 2:**
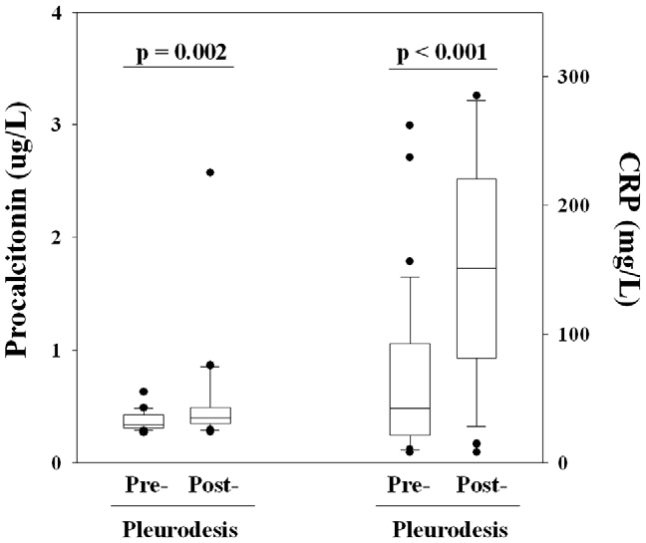
Changes in Procalcitonin and CRP before and after a non-infectious pro-inflammatory stimulus (talc pleurodesis). * The PCT values of three outliers (1 pre- and 2 post-pleurodesis) lied outside the margins of the graph and were not shown. N = 32 in each group (Wilcoxon Signed Rank test).

The second part of the study aim to further confirm that procalcitonin reflects only infective conditions and is not affected by non-infective inflammatory stimuli. Blood procalcitonin levels were measured before and at 24 to 48 hrs after talc pleurodesis, which is known to provoke intense pleural and systemic inflammation. The mean CRP level was significantly raised by 3.6 fold after pleurodesis, from 42 (22–90) to 151 (88–220) mg/L, *p*<0.001. In contrast, only a small and clinically insignificant increase (of 21%) in procalcitonin levels was observed: from 0.34 (0.31–0.42) to 0.41 (0.35–0.53) µg/L, *p* = 0.002 (Fig. 2).

### Role of procalcitonin in pleural infection

Patients with pleural infection (n = 214) were included, Table 2. A total of 102 patients with pleural infection who died or required surgery were first identified from the MIST cohort and matched (for age, gender and CRP) to controls who survived and did not require surgery, Table 2. Procalcitonin measurements failed in five of the 204 blood samples for technical reasons. The remaining 97 pairs of patients were included in the analyses.

**Table 2:** Procalcitonin levels in prediction of outcome of patients with pleural infection.

	Required Surgery and/or Death		Succesfully treated without surgery		*p* value *	
**Number**	97		97			
Died	53		0			
Surgery	44		0			
**Sex (Male∶ Female)**	70 ∶ 27		70 ∶ 27			
Died	34 ∶ 19					
Surgery	36∶ 8					
**Purulence of pleural fluid**	76 ∶ 21		83∶ 14			
Purulent ∶ Non purulent						
**Age**	62.7±16.4		62.4±16.0		p = 0.29	
Died (vs matched controls)	71.7±10.5		71.0±10.3		p = 0.09	
Surgery (vs matched controls)	51.9±15.8		52.0±15.7		p = 0.66	
**CRP**	178±105		176±108		p = 0.56	
Died (vs matched controls)	173±103		174±103		p = 0.93	
Surgery (vs matched controls)	184±108		177±115		p = 0.29	
**Procalcitonin** (ng/mL)	0.77 (0.48–1.51)		0.63 (0.45–1.35)		p = 0.25	
Died (vs matched controls)	0.80 (0.43–1.75)		0.61 (0.44–1.35)		p = 0.27	
Surgery (vs matched controls)	0.76 (0.48–1.39)		0.66 (0.47–1.37)		p = 0.66	

Patients who had died or required surgery were compared with patients with pleural infection who did not die or require surgery.

* paired t test or Wilcoxon signed rank test between case and matched controls.

**Figure 3:**
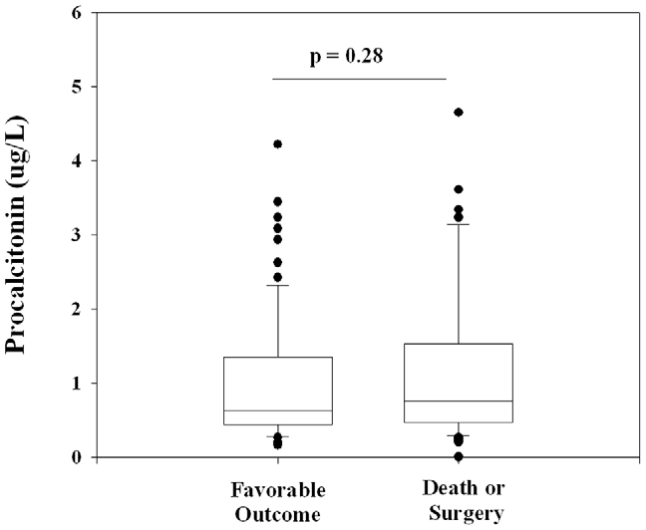
Procalcitonin levels were not significantly different between patients with favorable or poor outcomes matched for age, gender and CRP. N = 97 in each group; Wilcoxon Signed Rank test.

In this entire cohort of pleural infection patients (n = 214), procalcitonin measurements and CRP levels correlated poorly: R^2^ = 0.074, *p*<0.0001. The blood procalcitonin levels were not different between patients who died or required surgery and those who did not: 0.77 (0.48–1.51) vs 0.63 (0.45–1.35) µg/L respectively, *p* = 0.248 (Fig. 3). Subgroup analyses using surgery or death alone as separate endpoints also showed no significant differences in procalcitonin levels in subjects with adverse outcomes and the matched controls (Table 2).

**Figure 4:**
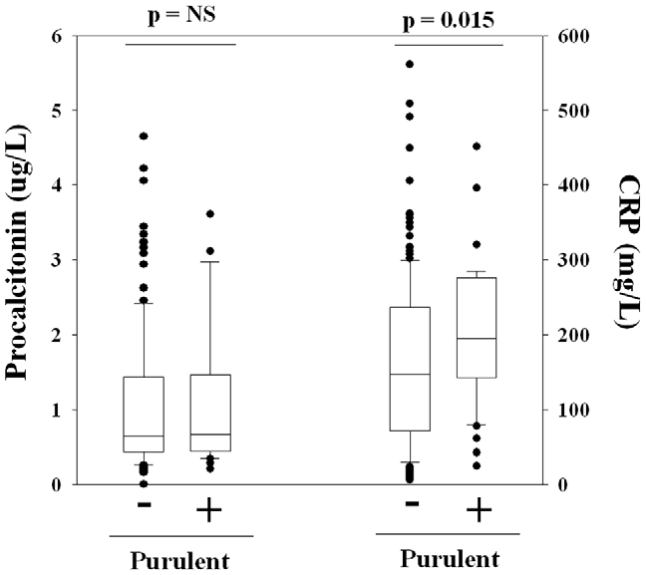
Procalcitonin and CRP levels between empyema patients with purulent (n = 41) and non-purulent pleural effusions (n = 174); Mann Whitney Ranked Sum test.

Comparing empyema patients with purulent (n = 41) and non-purulent (n = 174) pleural fluid, the median CRP was higher in the purulent group: 195 (145–274) vs 148 (73–236) mg/L, *p* = 0.015. However, there were no significant differences between the procalcitonin levels of the purulent and non-purulent groups: 0.67 (0.46–1.45) vs 0.65 (0.44–1.42) µg/L, *p* = 0.638 (Fig. 4).

## Discussion

The presented data show that procalcitonin reliably separate infective from non-infective causes even when adjusted for the degree of inflammation. Using unique clinical pleural settings, we were able to confirm that procalcitonin captures information specific to infection, rather than acting as an advanced inflammatory marker. Procalcitonin is the first peripheral blood biomarker that can help separate pleural infection from non-infective pleural diseases controlled for the severity of systemic inflammation. Procalcitonin level was significantly higher (by 1.7 fold) in empyema than in malignant effusions, matched for CRP, indicating that procalcitonin is superior to CRP for the identification of pleural infection. To provide further ‘proof of principle’, we showed that in patients with intense iatrogenic pleural inflammation following pleurodesis, procalcitonin remained relatively stable (∼20% rise) while the median CRP value rose by 360%.

Infection is inevitably accompanied by inflammation. Signs of infective and non-infective inflammation are often similar. Most conventional biomarkers (eg CRP and ESR) are surrogate markers of any inflammatory insult - infective or otherwise. Procalcitonin is a precursor of calcitonin and is released by the thyroid. The mechanism by which procalcitonin is upregulated during bacterial infection remains unclear [2], [3]. Use of procalcitonin has proved useful in many areas including the distinction between infective and non-infective cases of acute respiratory disease syndrome (ARDS) in intensive care settings [5], [18], between infective and sterile necrotizing pancreatitis [4], between bacterial and aseptic meningitis [19], between muscle inflammation and infection [20], etc. These comparisons are usually confounded by the differences in degree of inflammation between the infection group and the non-infective controls. Two recent studies showed that procalcitonin levels did not differ between patients treated with corticosteroids or not [21], [22]. This may serve as peripheral evidence that procalcitonin captures signals from infection and not inflammation. However, a definitive approach is needed to test if higher procalcitonin level truly reflects infection and not just more intense tissue inflammation.

The present study has addressed this question using two complimentary approaches in the unique setting of pleural effusions. Pleural effusions are common in clinical practice with over 50 causes identified [23]. Pleural inflammation underlies the pathogenesis of most exudative effusions; about 40% of which are parapneumonic or infection-related [24]. None of the current serum (eg CRP or ESR) or pleural fluid markers (eg LDH, protein, pH, cell counts) can reliably discriminate pleural infection reliably from non-infective (eg malignant, rheumatoid or drug) pleuritis [25].

In our first approach, we compared pleural infection patients with subjects with non-infective effusions but matched in systemic inflammation (using their blood CRP levels). Hence, the significant differences in procalcitonin levels of the two groups indicated that this marker can capture infective changes over and above CRP. In addition, procalcitonin was equally raised in non-purulent infective pleural fluid samples as in purulent ones. This will allow identification of infection in pleural fluid not immediately apparent as empyema.

In the second approach, we confirmed that procalcitonin is unaffected by non-infective inflammation using the unique opportunity of pleurodesis. Few clinical settings allow the deliberate provocation of an intense (local and systemic) inflammation; most such challenges can only be done in animal models. Pleurodesis, however, is a recognized therapeutic induction of aseptic pleural inflammation and has been shown to result in severe local and systemic responses. This is reflected in our study by the significant rise of CRP (by 360%) within 48 hours. Procalcitonin was distinctly superior to CRP, as unlike CRP, procalcitonin was minimally raised in the presence of inflammation without infection. These results provide much needed confirmation of the value of procalcitonin in correctly identifying infection from non-infective inflammatory diseases.

Recent studies have shown mixed results using procalcitonin to predict outcome of infection in different settings [1], [30]. Pleural infection is a significant illness in adults: ∼30% of patients failed conservative treatment (antibiotics and thoracostomy) and required surgery, or died [14]. In our study, a high procalcitonin level did not impact upon the eventual clinical outcome. Notably this is equally the case with CRP.

There are limitations to our study. This is intended to be a proof-of-concept study on the ability of procalcitonin to separate infection from non-infective inflammation, using two clinical cohorts of pleural disease patients. Although there are no obvious reasons why these results will not apply to inflammation of systems other than pleural diseases, it requires further evaluation. Pleural infection is predominantly caused by bacterial infection [26] and thus our conclusions on the advantages of procalcitonin cannot necessarily be extended to other microbial infections. Variations in performance of procalcitonin in infections from different organisms have been recognized [1]. The ROC values provided in the Results section were relevant only to our cohort of patients with pleural infection and pleural malignancies. It is not the aim of this study to establish the potential role, or cutoff values, of procalcitonin in the diagnostic algorithm for pleural effusions, which will require a large prospective study of consecutive patients with a broad range of pleural diseases.

Our data however lay solid evidence to justify future evaluation of procalcitonin in pleural diseases. Studies published to date on the use of procalcitonin in pleural diseases have been small, with different cutoff values and conflicting results [27]–[31]. The cost-effectiveness and the potential shortcomings of procalcitonin will need evaluation. Use of procalcitonin as a monitoring tool is a topical issue [32]; whether procalcitonin measurements can guide antibiotics use and/or surgical referral in pleural infection warrants examination.

No biomarkers can offer perfect sensitivity and specificity. Future studies should examine the role of procalcitonin, not in isolation, but when applied in conjunction with imaging, blood, pleural fluid and microbiological results. Procalcitonin is unlikely to function as a single diagnostic test but, based on this study, can offer important advantage over CRP in the workup of undiagnosed effusions.

In summary, this study provides important confirmation that procalcitonin is raised specifically during infection and can help discriminate infection from non-infective inflammatory conditions. Procalcitonin is superior to currently available tools, especially CRP, as a biomarker for infection, especially in pleural diseases.
